# Evaluation of a collagen matrix in a mandible defect in rats submitted to the use of bisphosphonates ^1^

**DOI:** 10.1590/s0102-865020200100000005

**Published:** 2020-11-30

**Authors:** Vanessa Vasconcelos Cunha, Paulo Goberlânio de Barros Silva, José Vitor Mota Lemos, Joyce Ohana Lima Martins, Milena Oliveira Freitas, Rafael Linard Avelar

**Affiliations:** I Fellow Master degree, Academic Master in Dental Sciences, School of Dentistry, Centro Universitário Christus (UNICHRISTUS), Fortaleza-CE, Brazil. Substantive scientific and intellectual contributions to the study.; II Professor, Division of Oral and Maxillofacial Surgery, School of Dentistry, UNICHRISTUS, Fortaleza-CE, Brazil. Critical revision, final approval.; III Dentist, School of Dentistry, UNICHRISTUS, Fortaleza-CE, Brazil. Statistics analysis, manuscript preparation.; IV Dentist, School of Dentistry, UNICHRISTUS, Fortaleza-CE, Brazil. Manuscript preparation.; V Dentist, School of Dentistry, UNICHRISTUS, Fortaleza-CE, Brazil. Acquisition, analysis and interpretation of data; technical procedures.

**Keywords:** Collagen, Osteonecrosis, Biphosphonates, Bone and Bones, Rats

## Abstract

**Purpose:**

To assess the effect of a collagen matrix (Mucograft^®^) on the inflammatory process in a semi-critical experimental defect model in rats treated with bisphosphonates.

**Methods:**

Eighteen Wistar rats were randomly divided into three groups: saline (CG), alendronate (ALD) 5mg/kg (AG) or zoledronic acid (ZA) 0.2mg/kg (ZG). ALD was administered orally for 10 weeks and ZA was administered intravascularly on days 0, 7 and 14 and 49. On day 42, a 2mm defect was created and filled with Mucograft^®^ collagen matrix. The contralateral side was filled with a clot (control side). The animals were euthanized 70 days after the beginning of the experiment and the hemimandibles were radiographically and histologically (counting of empty osteocyte lacunae (%), apoptotic (%) and total osteoclasts, neutrophil and mononuclear inflammatory cells) analyzed. The variables were submitted to ANOVA/Bonferroni and t test (parametric data) (p <0.05, GraphPad Prism 5.0).

**Results:**

Significant bone repair occurred in the groups treated with Mucograft^®^. High number of total inflammatory cells and neutrophils cells were showed in AG (p=0.026 and p=0.035) and AZ groups (p=0.005, p=0.034) on the control sides associated with delayed bone repair and the presence of devitalized bone tissue in AG and ZG on the Mucograft^®^ side.

**Conclusion:**

Mucograft^®^ collagen matrix attenuated the inflammatory process in a mandible defect in rats submitted to the use of bisphosphonates (AG and ZG).

## Introduction

Medication-associated osteonecrosis of the jaw (ONJ) is a serious complication in patients on drugs that inhibit bone resorption, such as bisphosphonates (BPs) and anti-RANKL monoclonal antibodies (i.e., denosumab ^1^ ^–^ ^3^ ). Since the first report in 2003 ^4^ , an increasing number of ONJ cases have been reported ^2^ .

Bisphosphonates are drugs that inhibit bone resorption, suppressing recruitment and activity of osteoclasts, thus reducing their useful life. These drugs are used as part of the chemotherapeutic treatment of bone cancers, such as multiple myeloma and metastatic disease in breast, prostate and lung cancer, which significantly impact patients’ quality of life ^5^ .

Despite their proven effectiveness as anti-resorptive drugs, a devastating side effect has been documented in data published in recent years ^3^ ^,^ ^6^ . Patients diagnosed with bisphosphonates-related osteonecrosis of the jaw (BRONJ) usually present alveolar bone exposure-necrosis, purulent secretion, pain, intraoral or extraoral edema and fistulas. Severe cases can lead to pathological fractures, oroantral fistulas and severe infections of the head and neck ^7^ .

The propensity to bisphosphonates-related osteonecrosis of the jaw (BRONJ) may be due to several anatomical and physiological factors. Bisphosphonates tend to be concentrated in the mandibular bone, and not in other skeletal sites, because they are placed in significant bone remodeling sites ^8^ ^–^ ^10^ . Thus, the forces of the masticatory function can easily induce microfractures that also require remodeling. In addition, unlike other skeletal sites, after surgery or trauma, the wound may be continuously exposed to more than 500 different species of microorganisms, resulting in high susceptibility to contamination and infection ^10^ .

One form of treatment of ONJ is to remove necrotic bone and apply surgical wound dressings ^2^ . One way to promote regeneration and cover exposed bone is to use collagen membranes such as Mucograft^®^. The type I and III porcine collagen membrane is a totally resorbable 3D matrix that promotes the proliferation of fibroblasts and induces the production of extracellular matrix ^11^ . According to Ramalingam *et al* . ^11^ histological studies have shown a decrease in the inflammatory infiltrate and absence of multinucleated giant cells.

However, in view of the pro-osteogenic properties of the Mucograft^®^ collagen matrix and possible use to control/treat ONJ, the objective of this study was to evaluate the effect of bone remodeling using the Mucograft^®^ collagen matrix in a model of osteonecrosis of the jaw induced by ALD and ZA

## Methods

This study was approved by the Animal Ethics Committee of the Centro Universitário Christus under protocol number 040/17 and performed in the laboratory of the same institution.

Eighteen Wistar rats ( *Rattus norvegicus* ) with a body mass between 180 and 220 grams were used. The animals were kept in appropriate cages, six animals per cage, individually numbered by a tail marking and kept in 12-hour light-dark cycles with water and food *ad libitum* .

### Experimental protocol

The animals were randomized and equally divided into the following three experimental groups: control group (CG), group treated with alendronate (AG) and group treated with zoledronic acid (ZG).

After anesthesia using xylazine (20mg/kg) and ketamine (80mg/kg), the animals in the control group were treated weekly with 0.1ml/kg of saline solution by gavage and intravenously. The animals in the alendronate group were treated weekly with 0.1 ml/kg of alendronate 70mg (EMS^®^, Brasilia-DF, Brazil), 6mg/kg by gavage and 0.1ml/kg of sterile saline solution (penile access). The animals in the ZG were weekly administered 0.1 ml/kg of sterile saline and 0.2 mg/kg of ZA (Eurofarma^®^, Itapevi-SP, Brazil) was administered intravascularly, in accordance with a previously published protocol [8]. In this group, 0.1ml/kg of ZA was administered on days 0, 7 and 14, 0.1ml/kg saline solution was administered on days 21, 28, 35 and 42 (day of the surgical procedure), 0.1ml/kg ZA was administered on day 49, and 0.1ml/kg solution saline was administered on days 54 and 63. The animals were weighed weekly to assess body mass gain (final mass: per initial mass x 100 %) ( Table 1 ). The animals were euthanized by an overdose of xylazine (50mg/kg) and ketamine (Syntec^®^, Santana de Paraiba-SP, Brazil) (150mg/kg) on day 70.

**Table 1:** Cellular profile of the mandible of rats treated with saline, ALD or AZ and the semi-critical mandibular defect model treated with or without Mucograft^®^.

		Experimental group			
		Saline	AG	ZG	p-V=value a
**Empty osteocyte lacunae (%)**					
	Control	13.2±0.5	42.9±4.3 *	46.8±10.7 *	0.025
	Mucograft^®^	11.0±2.3	48.1±5.6 *	41.1±5.3 *	0.003
	**p-value** ^b^	0.269	0.540	0.732	
**Osteoclasts (n)**					
	Control	2.0±1.5	2.7±1.2	1.8±0.7	0.842
	Mucograft^®^	2.0±0.9	2.5±0.5	3.0±1.7	0.829
	**p-value** ^b^	1.000	0.892	0.523	
**Apoptotic osteoclasts (%)**					
	Control	0.0±0.0	22.5±13.1	62.5±21.6 *	0.036
	Mucograft^®^	0.0±0.0	33.3±20.1	62.6±18.5 *	0.034
	**p-value** ^b^	1.000	0.722	1.000	
**Neutrophil polymorphonuclear**					
	Control	0.2±0.1	44.3±12.5 *	41.6±14.0 *	0.004
	Mucograft^®^	0.0±0.0	16.7±5.4 *	18.6±1.6 *	<0.001
	**p-value** ^b^	1.000	0.035	0.034	
**Mononuclear (n)**					
	Control	0.0±0.0	15.7±8.1	16.5±10.2	0.446
	Mucograft^®^	0.0±0.0	14.0±8.0	19.8±5.9	0.071
	**p-value** ^b^	1.000	0.925	0.777	
**Inflammatory cells (n)**					
	Control	0.2±0.1	86.7±23.7 *	85.0±17.3 *	<0.001
	Mucograft^®^	0.0±0.0	30.7±10.5 *	38.4±5.4 *	<0.001
	**p-value** ^b^	1.000	0.026	0.005	

a ANOVA/Bonferroni test;

student's *t* -test;

* p <0.05 *vs.* saline;

† p <0.05 *vs.* ALD (mean ± SEM) AG - alendronic group; ZG - zoledronic group.

The dose of ZA and ALD was obtained by converting the recommended dose for the treatment of bone metastases in humans as described by SILVA *et al* . ^8^ . Since the dose of alendronate recommended for the treatment of osteoporosis in an adult person weighing approximately 75 kg is 70 mg/week (0.95 mg/kg/week), as suggested by the Food and Drug Administration, the estimated dose for rats was 6 mg/kg/week (human dose (mg/kg)* 6.2).

### Surgical procedure

The surgery took place one month after the last dose of ZA (D42), under anesthesia with xylazine (20mg/kg) and ketamine (80mg/kg), following the protocol by for the creation of calvary defects, adapting it to the mandibular ramus.

**Figure 1:**
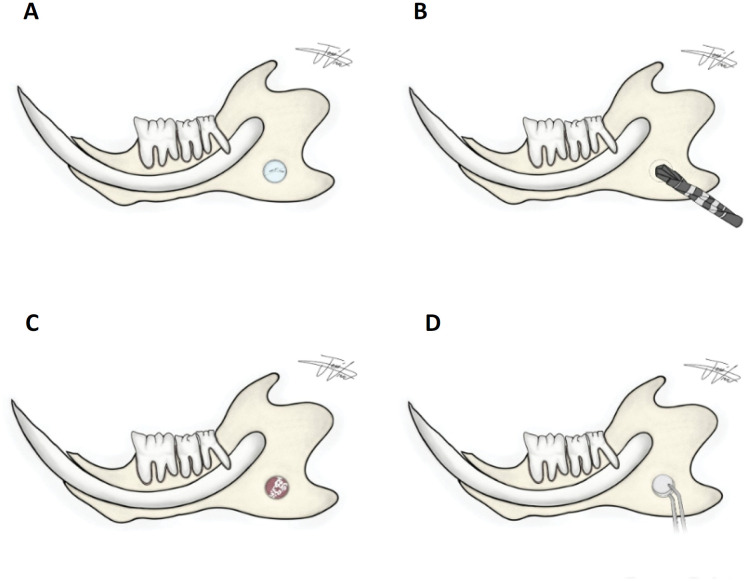
**A.** Schematic model of the semi-critical defect in the mandibular ramus of Wistar rats submitted to the administration of sterile saline, ALD or AZ. **B.** Schematic drawing of the drill throws into the bone defect. **C.** Drawing of the bone defect filled with a clot. **D.** Mucograft^®^ drawing on the bone defect.

After trichotomy was performed on the mandibular ramus, the dermis was disinfected using three applications of 1% chlorhexidine digluconate spray (Neba-Sept^®^, Panvel, Londrina-PR, Brazil), incision in the mandibular angle using a number 15 scalpel blade (Solidor^®^, Sao Paulo-SP, Brazil) on a scalpel handle (Bad Parker Golgran^®^, Sao Caetano do Sul-SP, Brazil) and the masseter muscle was dissected using Seldin Golgran^®^. After visualization of the mandibular ramus, a circular bicortical defect was prepared using a 2-mm drill (Neodent^®^, Straumann, Basel-Switzerland) coupled to a Neogrug XT Plus Neodent^®^ engine rotating at 1100 rpm for approximately 10 seconds under irrigation with saline water ( Fig. 1 ).

The defect was made bilaterally, and the left side was closed with a 4-0 suture needle (SK140, Procare^®^ Rio de Janeiro-RJ, Brazil) using the single-suture technique after filling it with an autogenous clot. The right side was filled with the Mucograft^®^ collagen matrix in the size corresponding to the surgical defect and closed with suture thread (needle thread for suture Procare^®^ Seda, 4-0, SK140) using the same technique. Morphine 0.25mg/kg was administered by gavage every 12 hours for three days to reduce postoperative pain.

### Digital radiographic analysis

**Figure 2:**
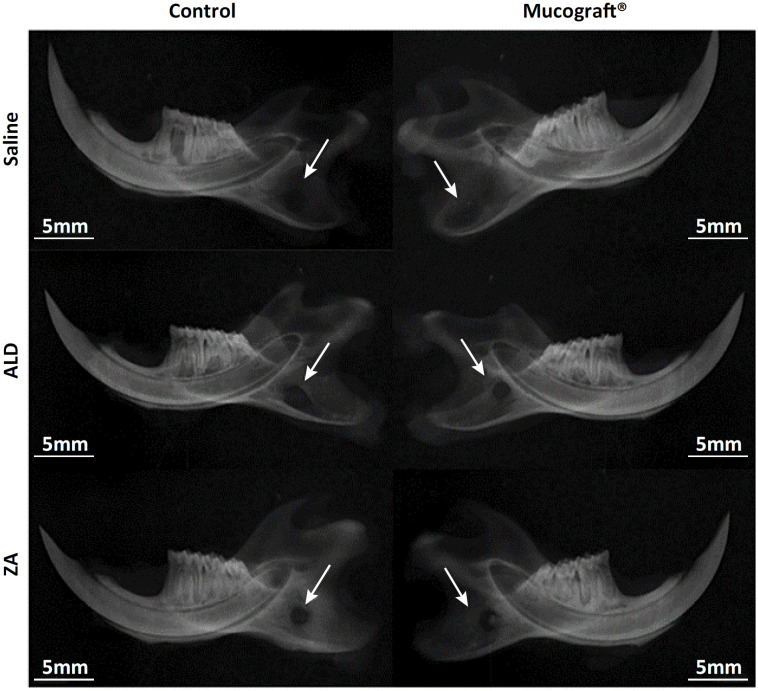
Radiographic analysis of the mandible of rats treated with saline, ALD or AZ and the semi-critical mandibular defect model treated with or without Mucograft^®^.

The hemimandibles of all groups were radiographed using a conventional X-ray device (63Kvp, 8mA, DabiAtlante^®^, Ribeirao Preto-SP, Brazil) coupled to the digital image capture device (Digora, Kavo^®^, Joinville-SC, Brazil). The parallel radiographic technique (long cone) used an SE localizer. The hemimandibles were positioned parallel to the radiographic film and the focus-film distance was 10 cm. Exposure time was established at 0.18 seconds, setting the function of the digital periapical radiograph for the upper anterior dental arch (13-23). The hemimandibles were qualitatively evaluated by three oral radiologists (kappa = 0.873) that investigated and described the aspects of healing and signs of BRONJ ( Fig. 2 ).

### Laminae and histopathological analysis

The hemimandibles were decalcified in 10% EDTA solution (pH 7.3; NaOH, PA) for 30 days and kept in suspension. After decalcification, the material was included in paraffin, cut at a thickness of 3μm, and stained with hematoxylin-eosin for further qualitative analysis using conventional light microscopy.

**Figure 3:**
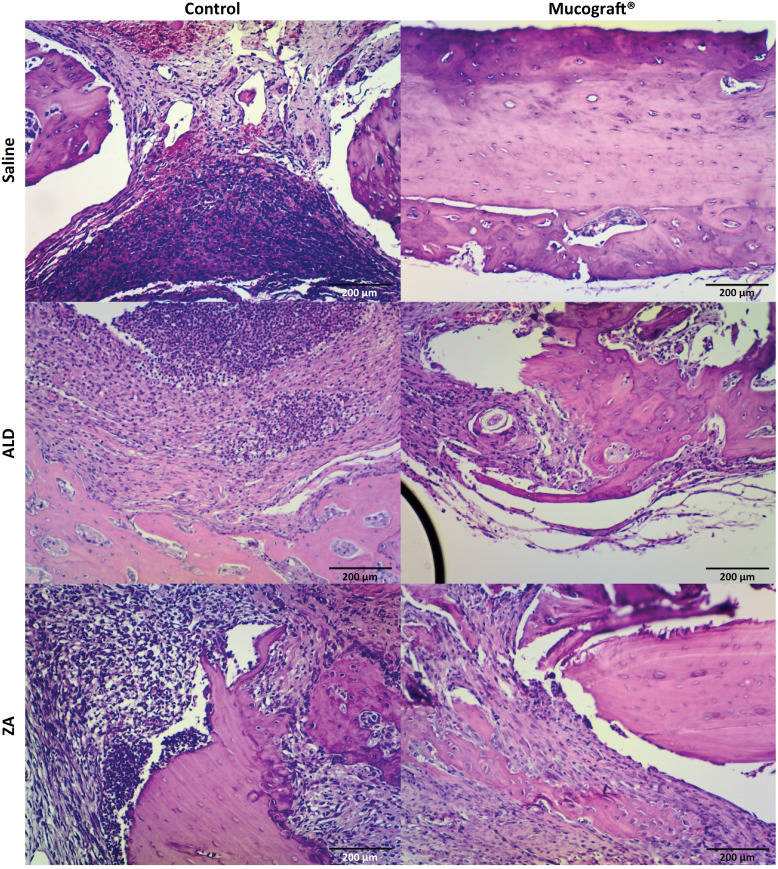
Microscopic analysis of the mandible of rats treated with saline, ALD or AZ and the semi-critical mandibular defect model treated with or without Mucograft^®^ (HE, x40). **Inflammatory infiltrate; VB: vitalized bone; NO: necrotic bone.

The lamina were qualitatively described and 10 fields were photographed at 400x magnification for subsequent counting of viable and empty osteocyte lacunae to calculate the percentage of empty osteocyte lacunae, viable and apoptotic osteoclasts to calculate the percentage of apoptotic osteoclasts, neutrophil polymorphonuclear and mononuclear cells ^8^ ( Fig. 3 ).

### Statistical analysis

The variables were submitted to Kolmogorov-Smirnov tests for normality, expressed as mean ± SD, and analyzed using one-way or two-way ANOVA followed by the Bonferroni post-test (parametric data) or Kruskal-Wallis followed by Dunn's post-test (non-parametric data). All analyses were performed using the GraphPad Prism^®^ 5.0 statistical software and the significance index adopted for all evaluations was p <0.05.

## Results

### Evaluation of body mass gain in rats undergoing mandibular defect model and treated with bisphosphonates

**Figure 4:**
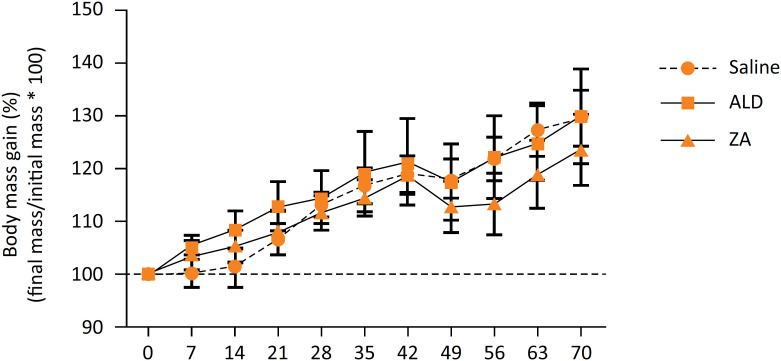
Body mass variation of rats treated with saline, ALD or AZ and the semi-critical mandibular defect model.

All animals presented body mass gain throughout the experiment. One week after the surgical procedure, there was a significant weight reduction on day 49, followed by weight gain until day 70 (p <0.001). There were no significant differences between the three experimental groups throughout the study (p = 0.740) ( Fig. 4 ).

*p <0.05, two-way ANOVA with repeated measures/Bonferroni test (mean ± SD).

### Evaluation of radiographic findings in rats undergoing mandibular defect model and treated with bisphosphonates

Radiographic analysis showed a diffuse radiolucent area in the control group with well-defined edges on the control side and total tissue repair on the Mucograft^®^ side. In AG, a radiolucent lesion with irregular edges in the mandibular ramus was observed on both sides, but smaller on the Mucograft^®^ side. A radiolucent lesion with irregular edges was observed on both sides in the ZG, but a discrete radiopaque marker was observed ( Fig. 3 ).

### Treatment with Mucograft^®^ modifies local inflammatory infiltrate profile; however, it does not reduce histological aspects of ONJ

Histologically, the results were similar. Total bone neoformation was observed in the CG treated with Mucograft^®^ when compared to the control side (empty osteocyte lacunae = 13.2±0.5% in saline and 11.0±2.3% in Mucograft^®^ sides). Inflammatory cells were absent in Mucograft^®^ (0.0±0.0 cells) and light in Control side (0.2±0.1). Intense inflammatory infiltrate was observed in the AG and ZG on the control sides (86.7±23.7 and 85.0±17.3 cells, respectively) associated with delayed bone repair and devitalized bone tissue (42.9±4.3% and 46.8±10.7% empty osteocyte lacunae, respectively).

**Figure 5:**
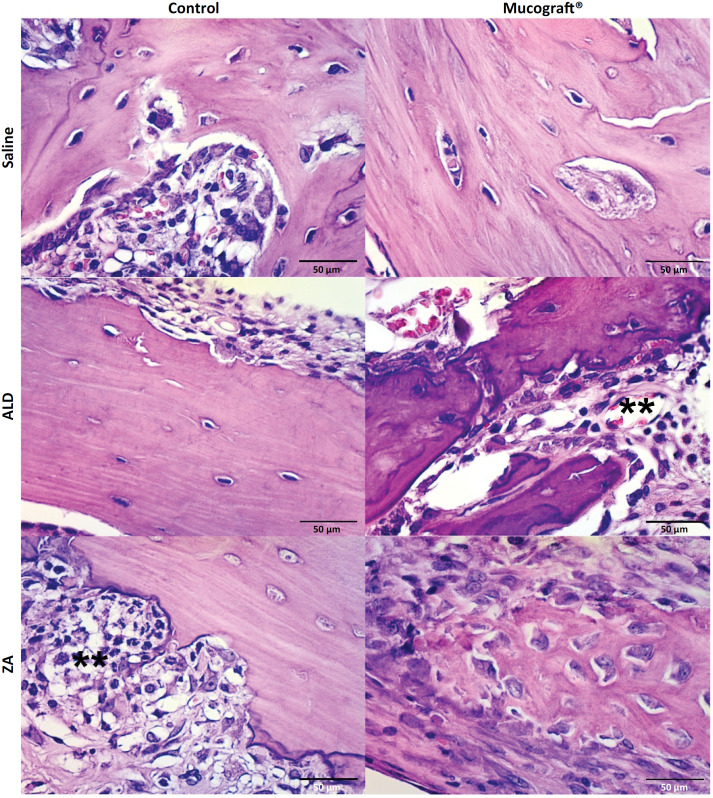
Microscopic analysis of the mandible of rats treated with saline, AZ or ALD, and critical mandibular defect model treated with or without Mucograft^®^ (HE, x400). **Inflammatory infiltrate.

On the Mucrograf^®^ side, a discrete inflammatory infiltrate (30.7±10.5 cells) was also associated with delayed bone repair and devitalized bone tissue, respectively. The use of the Mucograft^®^ collagen matrix accelerated bone repair in the CG and reduced imaging signs of ONJ in the AG (48.1±5.6% empty osteocyte lacunae) and ZG (41.1±5.3% empty osteocyte lacunae), without affecting delayed bone repair and ONJ, respectively ( Fig. 5 ).

The percentage of empty osteocyte lacunae was significantly increased in the AG (42.9±4.3%) and ZG (46.8±10.7%) when compared to the group treated with saline solution (13.2±0.5%), both for the animals in the control group (p = 0.025) and animals with the Mucograft^®^ collagen matrix (48.1±5.6%, 41.1±5.3% and 11.0±2.3%, respectively) (p = 0.003). There was no significant difference between the percentage of viable osteocyte lacunae between the Control and Mucograft groups treated with saline (p = 0.269), ALD (p = 0.540) and ZA (p = 0.732) ( Table 1 ).

The AG and ZG showed an increase in the number of polymorphonuclear cells when compared to the group treated with saline solution, both on the control side (p = 0.004) and the Mucograft^®^ side (p <0.001). However, on the Mucograft^®^ side, the number of polymorphonuclear cells was significantly lower in the animals treated with ALD (p = 0.035) and those treated with ZA (p = 0.034) ( Table 1 ).

There was no difference in mononuclear cell count between the experimental groups (p> 0.05). However, the AG and ZG showed a significant increase in the number of inflammatory cells in relation to the saline group on the control side (p <0.001) and the Mucograft^®^ side (p <0.001). Mucograft^®^ membrane implantation significantly reduced the number of inflammatory cells in the animals treated with ALD (p = 0.026) and those treated with ZA (p = 0.005) ( Table 1 ).

## Discussion

The pathophysiology of ONJ is multifactorial. The lack of treatment options highlights the medical and scientific inability to define the most prevalent causality. Bisphosphonates inhibit osteogenic cells, restricting angiogenesis, and have a negative effect on endothelial cells that compromises the healing of the oral mucosa ^10^ ^–^ ^12^ . Under these circumstances, the soft tissues are unable to cover the surgical wound, maintaining bone exposure, which worsens the inflammatory condition ^10^ ^–^ ^13^ . Thus, the present study was conducted to evaluate a material that could improve the inflammatory response under these conditions.

The two most potent bisphosphonates are AZ and ALD ^14^ . AZ is the treatment of choice for breast and metastatic prostate cancer and it is often associated with ONJ, which was used in this study due to its greater potency among bisphosphonates (20 times more potent than alendronic acid). Alendronate, on the other hand, is used orally to control and treat advanced osteoporosis and for that reason it was used as control and administered orally (by gavage) ^14^ ^–^ ^15^ .

In this study, we investigated the effect of a collagen matrix to improve angiogenesis, epithelialization, and formation of bone sequestration in bisphosphonate-induced osteonecrosis. Various materials in the form of membranes have been tested to promote bone healing in patients with ONJ. The use of a platelet-rich plasma (PRP) membrane was proposed in a surgical protocol to reduce the occurrence of osteonecrosis in patients undergoing treatment with IV bisphosphonate who required extraction ^15^ . Bocanegra-Perez *et al* . ^16^ observed accelerated angiogenesis around necrotic bone in rabbits combining vascular tissue and a single injection of PRP. In a study on rabbits, Aguirre and colleagues stated that the use of plasma rich in growth factors (PRGF) accelerates epithelialization and reduces inflammation in tongue wounds ^17^ . Our results using a collagen membrane showed similar results in which we observed a decrease in the inflammatory process with a decrease in polymorphonuclear cells in both groups where the collagen membrane was used.

One of the questions about PRP is the possibility of promoting infections because blood agar is used in microbiology to grow bacteria. Despite the similarity, PRP is not a different substrate from blood clots that naturally occur in wounds; thus, bacterial growth is expected to be similar to what occurs in any blood clot. In addition, the pH values of PRP vary between 6.5 and 6.7, comparatively more acidic than those of blood (7.0-7.2) and, therefore, we expect PRP to be less favorable for bacterial growth ^16^ . Another issue to be considered is the overexpression of growth factors and their receptors, associated with the tumor and in patients with dysplastic lesions, which suggests the possibility of inducing carcinogenesis or metastasis ^18^ . Therapeutic concentrates rich in growth factor could act as promoters (not initiators) of carcinogenesis, promoting the division and growth of mutant cells. However, it seems reasonable to avoid the use of PRP in patients with pre-cancerous oral lesions or with a history of oral squamous cell carcinoma. In these patients, based on the results, other types of materials such as the collagen membrane, which would decrease the inflammation present at the site, could be recommended.

Mucograft (MG) is a collagen matrix composed of type I and type III porcine collagen without chemical treatment. MG is bilayered with a thin and smooth compact layer composed of a low-porosity collagen framework and a porous layer with a noncompact, three-dimensional collagen framework. The efficacy of MG has primarily been assessed in terms of soft-tissue augmentation because of characteristics such as ease of handling, no requirement for preoperative hydration, and reduced chairside time ^19^ . Another histological finding revealed a favorable tissue reaction to MG with minimal inflammation and absence of multinucleated giant cells ^11^ , corroborating the findings in the present study of lower inflammation and the presence of multinucleated cells.

Based on the results, MG is an effective membrane that can be used in bone defects caused by osteonecrosis associated with bisphosphonates. The material is easy to use and manipulation does not present risk of infection and it can be used in association with other techniques.

## Conclusions

Mucograft is an effective membrane that can be used in bone defects caused by osteonecrosis associated with bisphosphonates. The material is easy to use and manipulate, has presented a minimal risk of infection and can also be used in association with other techniques.
